# Establishment of three heterogeneous subtypes and a risk model of low-grade gliomas based on cell senescence-related genes

**DOI:** 10.3389/fimmu.2022.982033

**Published:** 2022-08-16

**Authors:** Jing Chen, Lingjiao Wu, Hanjin Yang, XiaoChen Zhang, SuZhen Xv, Qiong Qian

**Affiliations:** ^1^ Department of Medical Oncology, The First Affiliated Hospital of College of Medicine, Zhejiang University, Hangzhou, China; ^2^ Collaborative Innovation Center for Diagnosis and Treatment of Infectious Diseases, State Key Laboratory for Diagnosis and Treatment of Infectious Diseases, The First Affiliated Hospital, College of Medicine, Zhejiang University, Hangzhou, China; ^3^ Department of Pathology, The First Affiliated Hospital of College of Medicine, Zhejiang University, Hangzhou, China

**Keywords:** low-grade glioma, cell senescence, tumor microenvironment, molecular subtypes, prognostic model

## Abstract

**Background:**

Cellular senescence is a key element in the occurrence and progression of a variety of tumors. As a result, cellular senescence-related markers can be categorized based on the prognosis status of patients. Due to the heterogeneity and the complexity of the tumor microenvironment (TME), the long-term effectiveness of low-grade glioma (LGG) treatment remains a clinical challenge. Consequently, developing and refining effective treatment approaches to aid with LGG management is critical.

**Methods:**

Based on the expressions of cell senescence-related genes (CSRGs) acquired from the cellAge database, consensus clustering was utilized to identify stable molecular subtypes. Clinical features, immune infiltration, route modifications, and genetic changes of various subtypes were also assessed. Following that, the least absolute shrinkage and selection operator (LASSO) regression and univariate Cox regression analysis were used for developing the cell senescence-related risk score (CSRS) model. Finally, a correlation study of the CSRS model with molecular, immunological, and immunotherapy parameters was performed.

**Results:**

C1, C2, and C3, are the three senescence-related subtypes that were identified. Patients belonging to the C1 subtype had poor prognoses and a substantial proportion of them was in the grade G3. The differentially expressed genes (DEGs) among the three subtypes were used to develop the CSRS model. In both the training and independent validation cohort, the model had a high area under the receiver operating characteristic (ROC) curve in predicting the overall survival (OS) of patients. As a result, this model can predict clinical features and responses to immunotherapy in a variety of patients and it is a potential independent prognostic factor for LGG.

**Conclusion:**

This research discovered three LGG subtypes related to cell senescence and created a CSRS model for six genes. Cell senescence was highly associated with unfavorable prognosis in LGG. The CSRS model can be used to predict the prognosis of patients and identify patients who would benefit from immunotherapy.

## Introduction

Low-grade glioma (LGG) is a common central nervous system tumor that typically consists of World Health Organization grades II and III and is less malignant than glioblastoma (GBM) (1). LGG has recently been shown to have molecular traits that can help with diagnosis and treatment. IDH1, IDH2, TP53, EGFR, and ATRX mutations, 1p/19q co-deletion, and MGMT promoter methylation are all known prognostic markers for LGG patients. These genetic characteristics, on the other hand, are unable to accurately predict survival outcomes. Despite advancements in LGG therapies such as surgical resection, adjuvant chemotherapy, postoperative radiation, and immunotherapy (2), patients with LGG still have a low overall survival rate. Therefore, studying the underlying molecular mechanisms of LGG initiation and progression for identifying effective biomarkers is crucial to optimizing LGG diagnosis and treatment regimes.

Cell senescence is a sustained proliferative arrest hallmarked by changes in cell shape, gene expression, heterochromatin formation, and metabolic activity caused by excessive stress-inducing stimuli (3). Following the identification of various cell senescence-related markers, cellular senescence has been detected in several malignancies in recent years. Cell senescence serves two purposes. On the one hand, because their proliferative capacity is reduced, senescent tumor cells can impede carcinogenesis (4). Furthermore, tumorigenic Ras expression is linked to the presence of senescent cells in diverse cancer lesions (5). In precancerous lesions, inactivation of tumor suppressors promotes cell senescence. Moreover, VO-OHpic, a phosphate and tension homology deleted on chromosome ten (PTEN) inhibitor, also promotes cell senescence and reduces carcinogenesis (6). On the other hand, senescent cells often have oncogenic properties. The senescence-associated secretory phenotype (SASP) has been observed, and it can affect the tumor microenvironment in both the autocrine environment and paracrine manner. In mammary epithelial cells, senescent human fibroblasts can induce the formation of precancerous and malignant mammary epithelial cells (7). The CXCR2 ligands GRO- and IL-8 can drive malignant melanocytes to develop by expressing high levels of CXC chemokine receptor 2 (CXCR2) (8, 9). Senescent stromal cells can aid cancer cell metastasis by promoting epithelial-mesenchymal transition (EMT) (10). As a result, cell senescence is important for tumor progression, tumor pathway modulation, and immunotherapeutic responses. As a result, identifying cell senescence-related genetic traits can aid in a more thorough investigation of the mechanisms underlying the link between LGG progression and cellular senescence. Several systems biology approaches are currently available for identifying biomarkers and constructing genetic signatures linked to the prognosis of patients with LGG. Tan et al. looked at immune-related genes in LGG and discovered six genetic markers that could help diagnose LGG and predict patient prognoses (11). Bai et al. examined N6-adenosine methylation (m6A) methylation-regulated genes in LGG and built a prognostic model based on their findings, to improve prognosis prediction accuracy in LGG patients (12). Using Cox regression analysis, Liu et al. created a ten-gene signature for LGG (13). Young people with LGG, on the other hand, have a terrible prognosis. As a result, more stable prognostic models, as well as particular markers, must be investigated.

In this research, we studied stable molecular subtypes according to cell senescence-related genes (CSRGs) by constant clustering and carried out a comparison of pathway and immune features among subtypes. Afterward, differential expression analysis and LASSO were used to find prognosis-related CSRGs. Moreover, we made a cell senescence-related risk score (CSRS) model that can help in the treatment of LGG and aid in developing personalized treatment strategies for affected people.

## Materials and methods

### Data collection and pre-processing

The LGG dataset (TCGA–LGG) was gathered from The Cancer Genome Atlas and comprised RNA sequencing (RNA-seq) data and clinical information from 506 samples (TCGA). The Chinese Glioma Genome Atlas (CGGA, http://www.cgga.org.cn/) was also retrieved to obtain “mRNAseq 693 (batch1)” and “mRNAseq 325 (batch2).” By combining two batches of RNA-seq data, a total of 408 LGG samples (CGGA cohort) were included in this study. Following that, the TCGA–LGG and CGGA cohorts were employed as the training and validation sets, respectively. In addition, the cellAge database (https://genomics.senescence.info/cells/) yielded 279 CSRGs.

### Molecular typing of CSRGs

To classify data into distinct kinds, consistency matrices were created using the ConsensusClusterPlus R package’s consistency clustering function (14). The samples’ molecular subtypes were determined using CSRG expression data. Then, using the “km” method and “canberra” as the metric distance, 500 bootstraps were run, with each bootstrapping operation involving 80 percent of the patients in the training set. To establish the molecular subtypes of the samples, the number of clusters was varied from 2 to 10, with the ideal number established by computing the consistency matrix and the consistency cumulative distribution function.

### Lasso Cox regression analysis

A shrinkage estimation algorithm is the Lasso method. It constructs a penalty function that decreases some coefficients while setting others to zero, resulting in a more refined model. As a result, it preserves the benefit of subset shrinking and is a biassed estimator for multicollinear data. As a result, it is possible to pick variables while estimating parameters, allowing it to better tackle the multicollinearity problem in regression analysis. The Lasso Cox regression was carried out in this work with the help of the R package glmnet (15).

### Construction and evaluation of the CSRS model

The coxph function in the survival R package (https://mran.microsoft.com/web/packages/survival/index.html) was used to perform a univariate Cox analysis of CSRGs in the TCGA–LGG and CGGA cohorts, yielding two sets of CSRGs closely linked to the prognosis of LGG patients, and the overlapping genes were chosen for further analysis with the criterion of P value less than 0.05. Then, across the three categories previously identified, differently expressed CSRGs were discovered. Lasso regression was used to minimize the number of genes to produce prognosis-related CSRGs. The MASS package’s stepAIC was applied to further compress the number of prognostic CSRGs. StepAIC starts with the most complicated model and removes one variable at a time to reduce the AIC, with a smaller AIC value indicating a better model that achieves a sufficient fit with fewer parameters. In addition, each patient’s CSRS was calculated using the following equation: CSRS=Σβi×Expi, where Expi is the level of gene expression of prognosis-related CSRGs and β is the Cox regression coefficient of corresponding genes. CSRS score was converted to z-score. We set z-score = 0 as a cut-off to classify samples into high- (z-score > 0) and low-risk (z-score < 0) groups. Furthermore, the Kaplan–Meier (KM) algorithm was utilized for plotting the survival curves for subsequent prognostic studies. Finally, we used a log-rank test for determining the value of differences.

### Single-sample gene set enrichment analysis

The R package GSVA (16) was used to perform a single-sample gene set enrichment analysis (ssGESA) on the gene expression profiles corresponding to LGG samples in the TCGA–LGG cohort to examine the association between CSRS and biological functions in various samples. The scores of each sample on various functions were then measured (i.e., ssGSEA scores for each sample corresponding to each function). Finally, we calculated the correlations between these functions and CSRS.

### Patient response to different immunotherapies and drugs

To predict the clinical responsiveness of patients in the high- and low-CSRS groups to immune checkpoint inhibitors, the Tumor Immune Dysfunction and Exclusion (TIDE) algorithm was utilized (17). The TIDE algorithm probed into the M2 subtype of cancer-associated fibroblast (CAF), myeloid-derived suppressor cells (MDSCs), and tumor-associated macrophages as three cell types that reduced T-cell infiltration in cancers (TAMs). To avoid immune evasion, this algorithm used two different mechanisms: a malfunction score for tumor-infiltrating cytotoxic T cells (CTLs) and an exclusion score for the immunosuppressive factor CTL. Immune escape is more likely with a higher TIDE prediction score, implying that patients are less likely to benefit from immunotherapy. Moreover, we measured the half-maximal inhibitory concentration (IC50) of the drug using the pRRophetic R package (18) to observe the sensitivity of patients in the high- and low-CSRS groups to different chemotherapeutic agents and targeted drugs.

### Gene set enrichment analysis

In distinct biological processes, GSEA can reveal pathways of various molecular subtypes. GSEA was used in this investigation with all candidate gene sets from the Hallmark database (19), and FDR<0.05 was set as the criterion of substantial enrichment.

### Cell abundance in TME

The relative abundance of 22 immune cells in LGG was quantified using the CIBERSORT algorithm (20)(https://cibersort.stanford.edu/). The fraction of immune cells was also determined with the help of the Estimation of STromal and Immune cells in MAlignant Tumours Using Expression Data (ESTIMATE) software (21).

### Statistical analysis

R (https://www.r-project.org/, version 3.6.3) was used for all statistical studies and data visualization. P < 0.05 represented a significant difference, and all estimated P-values were two-tailed.

## Results

### Identification of three cell senescence-related molecular subtypes of LGG

**Figure 1:**
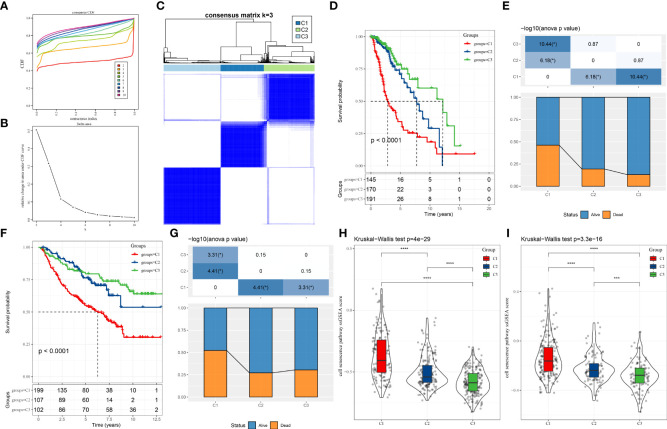
LGG subtypes sorted by CSRGs found in the TCGA–LGG and CGGA cohorts. **(A)** CDF curves of TCGA–LGG cohort samples. CDF curves for consensus scores (based on different numbers of subtype, k = 2 – 10) are illustrated using various colors; **(B)** CDF Delta area curves for samples in the TCGA–LGG cohort; **(C)** Clustering plot of consensus scores for samples in the TCGA–LGG cohort at k = 3; **(D)** KM curves indicating prognostic differences between the three molecular subtypes in the LGG cohort; **(E)** Differences in survival status of patients from different subtypes in the TCGA–LGG cohort; **(F)** KM curves indicating prognostic differences between the three molecular subtypes in the CGGA cohort; **(G)** Differences in survival status of patients from different subtypes in the CGGA cohort. **(H)** Differences in CSRS between the three molecular subtypes in the TCGA–LGG cohort; **(I)** Differences in CSRSs between the three molecular subtypes in the CGGA cohort. Significance was measured by variance analysis (*P < 0.05; ***P < 0.001; ****P < 0.0001).

Initially, we carried out a univariate cox analysis (P < 0.05) on CSRGs from both TCGA–LGG and CGGA datasets to get 115 genes strongly linked to LGG patients’ prognoses. Afterward, by consistent clustering, we grouped 506 LGG samples. Cluster number was optimized using the cumulative distribution function (CDF), and the CDF delta area curve suggested that the outcomes of clustering were stable when the number of clusters was 3 (**Figures 1A, B**). Consequently, the number (k) was selected as three to get three molecular subtypes (**Figure 1C**). We further studied the prognostic features of these three molecular subtypes. We observed a remarkable difference in patient prognosis between the three molecular subtypes in the TCGA–LGG cohort (**Figure 1D**), the best prognosis was observed in patients of the C3 subtype and patients of the C1 subtype showed the worst prognosis. Moreover, the mortality of patients in the C1 subtype was greatly enhanced in comparison with those in the C3 subtype (**Figure 1E**). Afterward, using the same strategy, molecular typing was carried out on samples in the CGGA cohort and we observed similar a remarkable difference in the prognosis of patients belonging to these three molecular subtypes (**Figures 1F, G**), which aligned with the outcomes from the TCGA–LGG training set. Then, a comparison was done between the CSRSs in the various molecular subtypes in the TCGA–LGG and CGGA cohorts (**Figures 1H, I**). Remarkable differences were observed in CSRSs of various molecular subtypes, the lowest CSRS was observed in the C1 subtype and the highest in the C3 subtype.

### Differences in clinicopathological characteristics among three molecular subtypes

The TCGA dataset was used for comparing the differences in clinical features among the three subgroups. There was no discernible gender difference between the three categories. However, the C1 subtype had a higher number of patients in the G3 grade, whereas the C2 and C3 subtypes had a higher proportion of patients in the G2 grade. In terms of IDH mutations, the C2 and C3 subtypes had the highest frequency of patients with mutations. Furthermore, the C3 subtype had a considerably larger number of individuals with 1p19q co-deletion than the C1 and C2 subtypes. Individuals with the C2 and C3 subtypes also had considerably more MGMT promoter methylation events than patients with the C1 subtype (**Figure S1A**). Patients who experienced both IDH mutations and 1p19q co-deletion also had the greatest prognosis, with a median OS of 8 years. Patients with an IDH mutation but no 1p/19q deletion (astrocytoma) had a median survival time of 6.4 years. Furthermore, patients with IDH wild-type LGG had a median OS of 1.7 years, which was comparable to those with IDH wild-type glioblastoma and commensurate with the prognosis of patients with the C3 subtype. In the CGGA cohort, differences in age, gender, grade, IDH mutation, 1p19q co-deletion, and MGMT promoter methylation were compared (**Figure S1B**). In the CGGA cohort, differences in age and gender were not significant.

### Differences in mutational characteristics among three molecular subtypes

**Figure 2:**
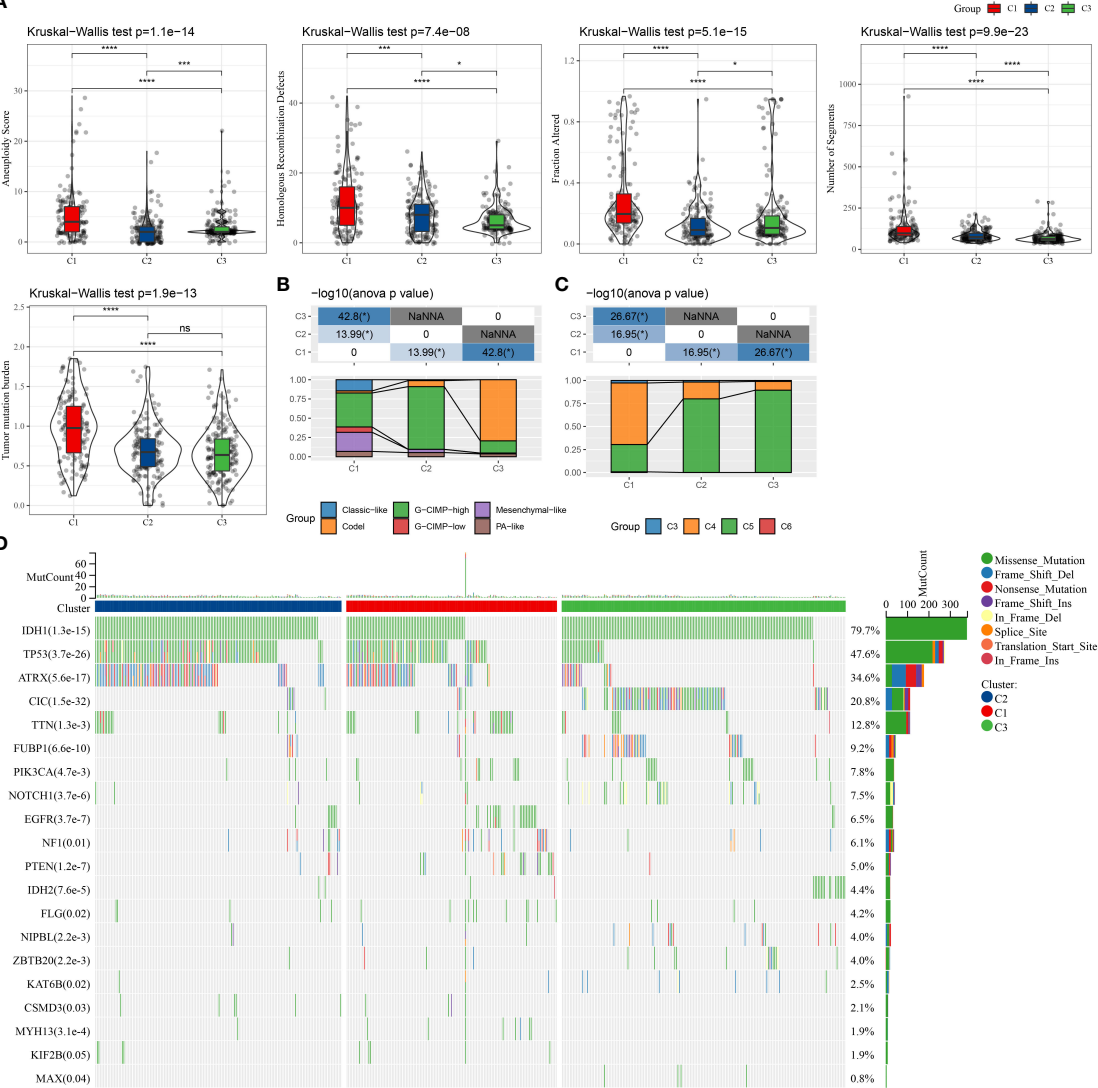
Comparison of genomic alterations among the three molecular subtypes. **(A)** Differences in fraction altered, the number of segments, homologous recombination defects, aneuploidy score, and tumor mutation burden in the molecular subtypes in the TCGA–LGG cohort; **(B)** Comparison of the three molecular subtypes with immuno-molecular subtypes; **(C)** Comparison of the three molecular subtypes with other molecular subtypes; **(D)** Somatic mutations in the three molecular subtypes. (ns, no significance. *P < 0.05, ***P < 0.001, ****P < 0.0001).

We analyzed the mutational profiles of various molecular subtypes further for revealing the possible underlying mechanisms used in the classification of cell senescence-related subtypes. In this report, data on the molecular properties in the TCGA–LGG cohort was retrieved from previous research on pan-cancer (22). The cellular senescence subtypes were linked with measures of DNA damage, such as the fraction of genome altered, homologous recombination defects, aneuploidy, tumor mutation burden, and the number of segments. Moreover, patients of the C3 subtype had lower scores of aneuploidies, number of segments, fraction altered, homologous recombination defects, and tumor mutation burden (**Figure 2A**). Additionally, further molecular subtypes were provided in the above study. Therefore, these six molecular subtypes were compared with our three molecular subtypes (**Figure 2B**). More “Codel” molecular subtypes were discovered in the C3 subtype and more “G-CIMP-high” molecular subtypes in the C2 subtype. Furthermore, LGG was sorted into six molecular subtypes based on 160 different immune signatures in the above study, the best prognosis was observed in patients of immunoassay subtype C3 and the worst prognosis was seen in subtypes C4 and C6. Hence, a comparison of these six immuno-molecular subtypes was carried out with the three molecular subtypes. We defined and observed that the C4 subtype of the immuno-molecular subtypes occupied more of the C1 subtypes (**Figure 2C**). Additionally, the connection between gene mutations and molecular subtypes was studied and a strong correlation was identified. In LGG, ATRX, CIC, IDH1, TP53, and TTN genes went through numerous somatic mutations. The IDH1 gene among them had a higher frequency of mutations in C2 and C3 subtypes, and patients with IDH1/2 mutations showed a better prognosis. Moreover, the TP53 gene had the highest mutation frequency in the C subtype, followed by the C1 subtype (**Figure 2D**).

### Differences in immune characteristics among three molecular subtypes

**Figure 3:**
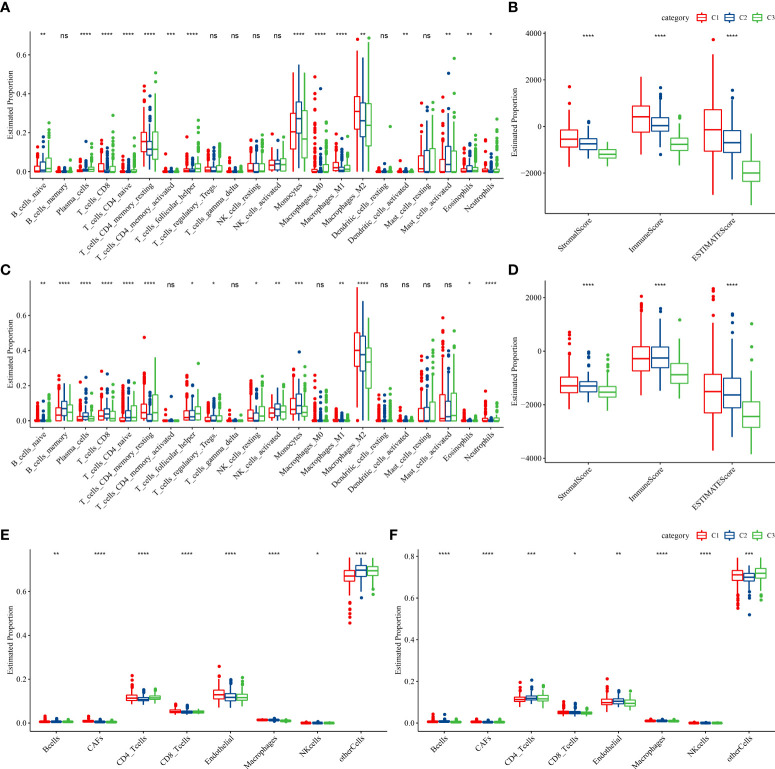
Proportions of immune cell components in the two LGG cohorts. **(A)** Differences in 22 immune cell scores among different molecular subtypes in the TCGA–LGG cohort; **(B)** Differences in ESTIMATE immune infiltration in various molecular subtypes in the TCGA–LGG cohort; **(C)** Differences in 22 immune cell scores in various molecular subtypes in the CGGA cohort; **(D)** Differences in ESTIMATE immune infiltration among various molecular subtypes in the CGGA cohort. **(E, F)** EPIC analysis for the estimated proportion of immune cells in TCGA-LGG **(E)** and CGGA **(F)** cohorts. (ns, no significance. *P < 0.05, **P < 0.01, ***P < 0.001, ****P < 0.0001).

To better understand the differences in the immunological milieu of patients belonging to distinct molecular subtypes, the degree of immune cell infiltration of patients in the TCGA–LGG cohort was measured using the expression levels of genes in immune cells. CIBERSORT was used to calculate the relative abundance of 22 immune cell types (**Figure 3A**), and most immune cell subtypes differed significantly. Immune cell infiltration was measured using ESTIMATE (**Figure 3B**), and patients belonging to the C1 subtype had a considerably higher “ImmuneScore” and immune cell infiltration degree than patients belonging to other subtypes. Finally, the immune infiltration degree of samples in the CGGA cohort was examined (**Figures 3C, D**), and a similar phenomenon was observed as in the TCGA cohort. Moreover, EPIC analysis also displayed the similar result with CIBERSORT analysis (**Figures 3E, F**)

### Pathway analysis of different molecular subtypes

**Figure 4:**
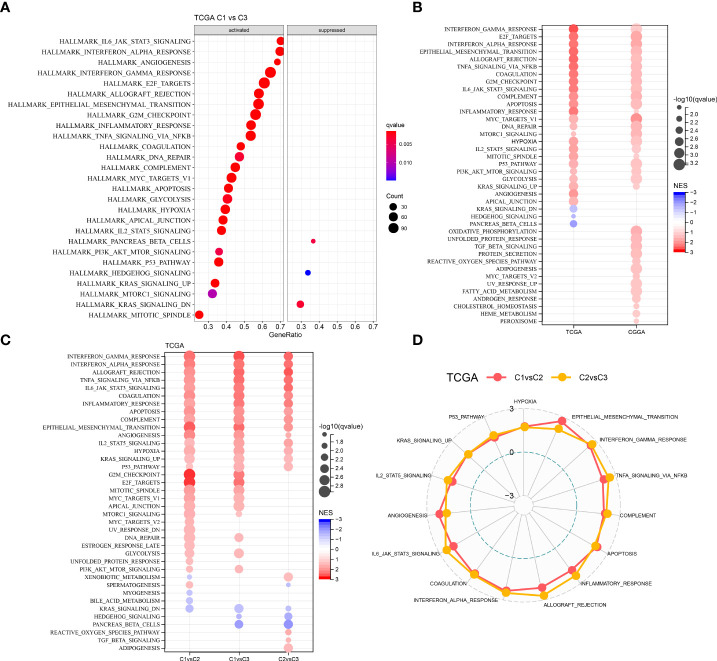
Comparative analysis of pathways between the three different molecular subtypes. **(A)** GSEA outcomes of C1 *vs* C3 in the TCGA–LGG cohort; **(B)** Bubble plots of GSEA outcomes of C1 *vs* C3 in two LGG cohorts; **(C)** Bubble plots of comparative outcomes between various molecular subtypes in the TCGA–LGG cohort; **(D)** Radar plot of consistently activated pathways in the TCGA–LGG cohort (C1 *vs* C2 and C2 *vs* C3).

We performed GSEA on all candidate gene sets from the Hallmark database (19) to find out the differentially activated pathways (DAPs) in different molecular subtypes. The C1 subtype was considerably enriched in 27 DAPs in the TCGA cohort, while 35 DAPs were significantly enriched in the CGGA cohort (**Figures 4A, B**). In addition, in different LGG cohorts, aberrant routes between C1 and C3 subtypes were compared (**Figure 4B**). Immune-related pathways such as interferon-gamma, interferon-alpha, allograft rejection, and inflammatory response were the most common DAPs. E2F targets, G2M checkpoint, and Myc targets v1 were also active, as were some cell cycle-related pathways (**Figure 4C**). Following that, DAPs between C1 and C2, C1 and C3 subtypes, and C2 and C3 subtypes in different TCGA–LGG cohorts were compared (**Figure 4D**). Immunomodulatory pathways, cell cycle-related pathways, and numerous critical tumor-related pathways, including P53, hypoxia, and EMT, were all active in patients with the C1 subtype. As a result, CSRGs might have an important role in both the immunosuppressive and malignant microenvironments (TME).

### Identification of DEGs associated with cell senescence-related subtypes

CSRGs were used to identify three separate molecular subtypes that were significant in the univariate analysis. Following that, with the criterion of FDR < 0.05 and |log2FC| > 1, the limma package was utilized for calculating the differentially expressed CSRGs (DECSRGs) across C1 and non-C1, C2, and non-C2, and C3 and non-C3 molecular subtypes. By looking for DECSRGs in different molecular subtypes, a total of 21 genes were discovered. For gene number reduction in the risk model, Lasso regression was employed to compress these 21 CSRGS even more. The number of independent variables whose coefficients tended to zero gradually rose as the lambda increased, as illustrated in **Figure S2A**, and the number of independent variables whose coefficients tended to zero gradually increased as the lambda increased. The confidence intervals under each lambda were assessed after the model was built using 10-fold cross-validation (**Figure S2B**). When lambda = 0.0317, the model was at its best. As a result, the target genes for the subsequent analyses were chosen from a list of eight genes with lambda = 0.0317. We also used the Akaike information criterion (AIC) to run a stepwise multivariate regression analysis based on these eight genes. AIC considers the model’s statistical fit as well as the number of parameters required to fit it. The MASS package’s stepAIC technique starts with the most complicated model and removes one variable at a time to reduce the AIC, with a smaller AIC value indicating a better model that achieves a sufficient fit with fewer parameters. Finally, six genes were identified as prognosis-related CSRGs: thymosin beta 4 (TMSB4X), cyclin-dependent kinase 6 (CDK6), forkhead box M1 (FOXM1), insulin-like growth factor-binding protein-5 (IGFBP5), integrin beta 4 (ITGB4), and IGFBP3 (**Figure S2C**).

### Construction and validation of the clinical prognostic model

**Figure 5:**
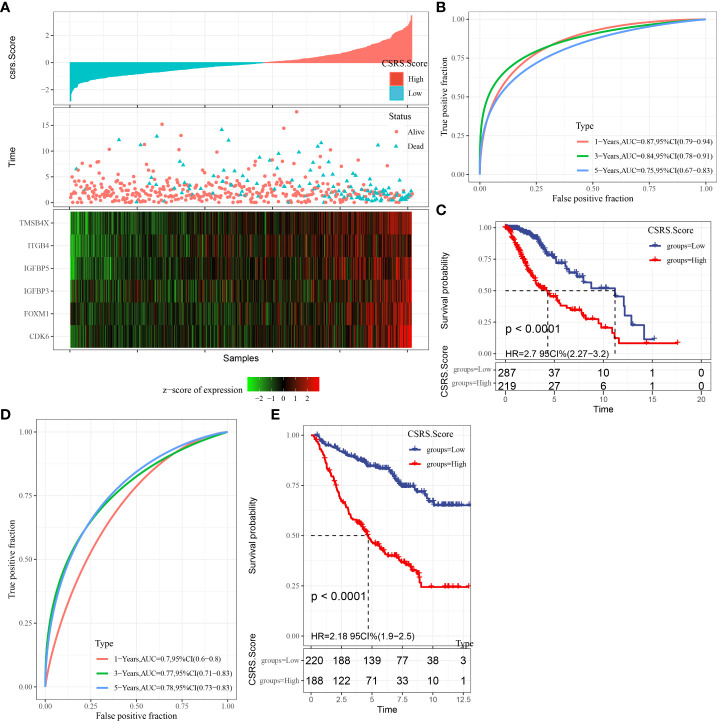
The creation and validation of the clinical prognostic model. **(A)** CSRS, survival time, survival status, and CSRG expression in the TCGA–LGG dataset; **(B)** ROC curves and AUC of CSRS in the TCGA–LGG dataset; **(C)** KM survival curves of CSRS in the TCGA–LGG dataset; **(D, E)** ROC curves and KM survival curves of CSRS in the CGGA cohort.

We used the expression levels and coefficients of six CSRGs to develop a prognostic model related to cellular senescence. Each sample’s CSRS was measured and normalized based on the CSRS calculation equation. Afterward, the samples were sorted into high- and low-risk (CSRS) groups as per the normalized cutoff value (0). The CSRS distribution of patients in the TCGA–LGG cohort is illustrated in **Figure 5A**. The mortality rate of patients in the high-risk group was high with a shorter survival time. Consequently, the worse prognosis of patients was related to high CSRSs. Furthermore, six genes had greatly increased expression levels with increasing CSRSs. Furthermore, ROC analysis for prognostic classification was done with the help of R package timeROC (23) and quantified the one-, three-, and five-year prognostic predictive effectiveness (**Figure 5B**), and the model had high AUC values (one-, three-, and five-year AUC values of 0.87, 0.84, and 0.75, respectively). Finally, the KM curve indicated a significant difference in survival between patients in the high- and low-CSRS groups (P < 0.0001), showing that the overall survival of patients having higher CSRSs was worse in the training cohort (**Figure 5C**). In addition, a validation analysis was done in the CCGA cohort to confirm the strength of the CSRS model. The CSRSs of patients in the CCGA cohort were identified similarly and the analysis outcomes are demonstrated in **Figures 5D, E**. Similar outcomes were seen in the validation cohort, patients with high CSRSs had a poor prognosis, and patients with low CSRSs had a better prognosis (P < 0.0001).

### CSRS distribution in different clinicopathological characteristics and patient prognosis

The CSRS distribution in the TCGA–LGG cohort was examined amongst different groups. Grade, IDH Mutation, IDH/codel subtype, and MGMT promoter methylation all showed significant differences in CSRS score in both two cohorts (**Figures S3A, B**). We also looked at the differences in CSRS between molecular subtypes, finding that patients with the C1 and C3 subtypes had the highest and lowest CSRS, respectively. The prognostic difference between our established high- and low-risk categories in the TCGA–LGG cohort was also evaluated, with the results indicating that our risk groupings were reliable (**Figure S3C**).

### Differences in immune/pathway characteristics between different SRS groups

**Figure 6:**
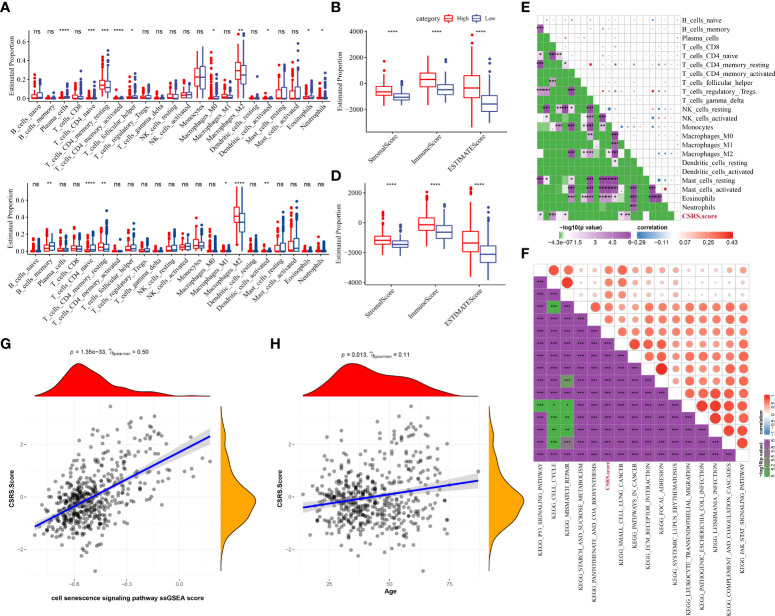
Differences in immune/pathway properties between different CSRS groups. **(A)** Proportion of immune cells in the TCGA–LGG cohort; **(B)** Proportion of immune cells in the CGGA cohort; **(C)** Proportion of immune cells in the TCGA–LGG cohort measured using the ESTIMATE software; **(D)** Proportion of immune cells in the CGGA cohort calculated using the ESTIMATE software; **(E)** Correlation analysis of 22 immune cells with cellular CSRS in the TCGA–LGG cohort; **(F)** Correlation analysis outcomes of KEGG pathways with a correlation coefficient greater than 0.6 with CSRS; **(G)** Correlation analysis of CSRS with prognosis-related CSRS in the TCGA–LGG cohort; **(H)** Correlation analysis of age with prognosis-related CSRS in the TCGA–LGG cohort. (ns, no significance. *P < 0.05, **P < 0.01, ***P < 0.001, ****P < 0.0001).

To better understand the changes in the immunological milieu, researchers analyzed the relative abundance of 22 immune cells in the TCGA–LGG cohort’s high- and low-CSRS groups (**Figure 6A**). The relative abundance of immune cells in the two groups differed significantly. ESTIMATE was also used to measure immune cell infiltration (**Figure 6B**). Patients with a high CSRS had considerably greater “ImmuneScore” and levels of immune cell infiltration than those with a low CSRS. Similar findings were also reported in the CGGA cohort (**Figures 6C, D**). The link between CSRS and 22 immune cells was then investigated (**Figure 6E**). CSRS was found to have a remarkable association with B cell naive, plasma cells, naive CD 4 T cells, M0 macrophages, and M1 macrophages. Moreover, we performed ssGSEA for calculating the correlation coefficient of these pathways with CSRS (**Figure 6F**) and filter out the pathways with a correlation coefficient greater than 0.6. Most of these pathways, like the p53 signaling pathway, JAK-STAT signaling pathway, and ECM receptor interaction had a positive relationship with CSRS. Moreover, a major positive correlation was observed between CSRS and necroptotic score (P = 1.35e-33, R = 0.5) (**Figure 6G**). Finally, the link between the age of the patients and CSRS was measured and a major positive association was observed between CSRS and age (P =0.013, R = 0.11) (**Figure 6H**).

### Differences in immunotherapy/chemotherapy efficacy between different CSRS groups

**Figure 7:**
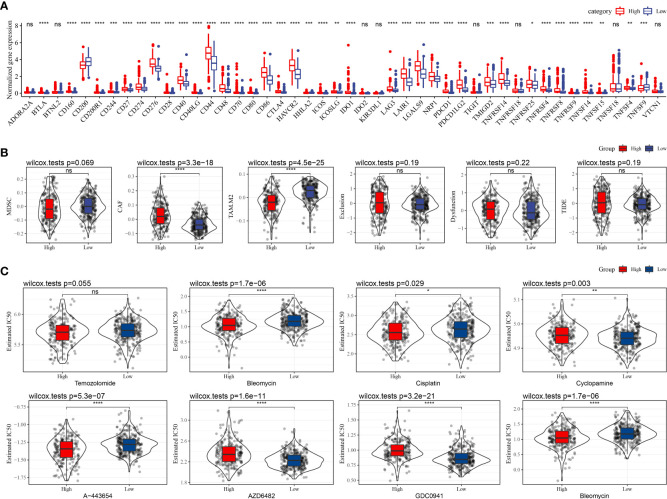
Differences in immunotherapy/chemotherapy effectiveness among two different CSRS groups. **(A)** Differentially expressed immune checkpoints between two CSRS groups in the TCGA–LGG cohort; **(B)** Differences in TIDE analysis outcomes among two CSRS groups in the TCGA–LGG cohort; **(C)** Box plots of the estimatedIC50 for Temozolomide, Bleomycin, Cisplatin, Cyclopamine, A-443654, AZD6482, GDC0941, and Bleomycin in TCGA–LGG cohort. (ns, no significance. *P < 0.05, **P < 0.01, ***P < 0.001, ****P < 0.0001).

The differences in immunotherapy sensitivity across patients in different CSRS groups in the TCGA–LGG cohort were investigated further. The difference in immune checkpoint expression between the two CSRS groups was first compared (**Figure 7A**). The expression of most immune checkpoint genes differed between the two CSRS groups. Immune checkpoint gene expression was found to be considerably higher in the high-CSRS groups than in the low-CSRS groups. Following that, the immunotherapy efficacy differences between the two CSRS groups were compared. The TIDE program was used to evaluate the clinical effects of immunotherapy on the two categories of patients. No significant differences in MDSC, dysfunction, exclusion, or TIDE scores were found in the TCGA–LGG cohort, as illustrated in **Figure 7B**. The response of patients in the two CSRS groups in the TCGA–LGG cohort to traditional chemotherapy medications such as Temozolomide, Bleomycin, Cisplatin, Cyclopamine, and Bleomycin, as well as targeted therapies such as A-443654, AZD6482, and GDC0941, was also studied. Cisplatin, A-443654, and Bleomycin were more sensitive in the high-CSRS group, whereas AZD6482, Cyclopamine, and GDC0941 were more sensitive in the low-CSRS group (**Figure 7C**).

### CSRS–nomogram improves the accuracy of patient prognosis and survival prediction

**Figure 8:**
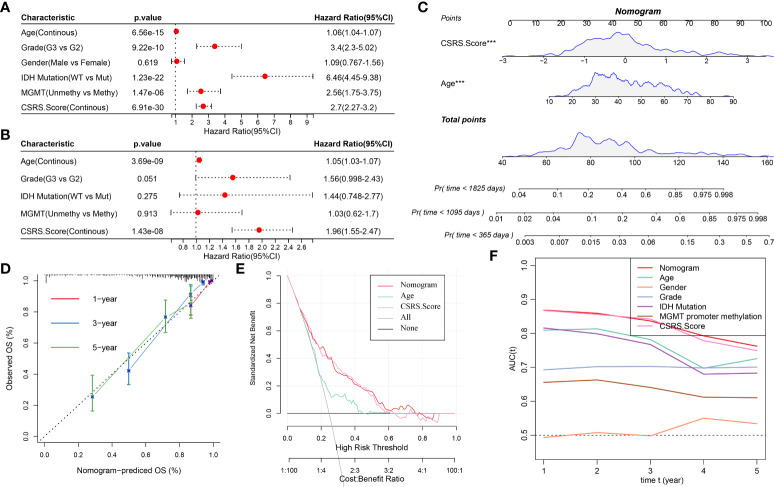
Developing the nomogram according to CSRS. **(A, B)** Univariate and multivariate Cox analysis of CSRS and clinicopathological properties; **(C)** Constructing a Nomogram model; **(D)** One-, three-, and five-year calibration curves for the nomogram; **(E)** Decision curve for the nomogram; **(F)** Prognosis-related AUC predicted by different clinical variable ***P < 0.001.

Univariate and multivariate Cox regression analyses of CSRS and clinicopathological features in the TCGA–LGG cohort revealed that CSRS was the most important prognostic predictor, with age being an independent prognostic factor (**Figures 8A, B**). Following that, a nomogram including CSRS and age was created (**Figure 8C**). The most significant impact on survival prediction was CSRS. The calibration curve was used to further assess the model’s prediction accuracy (**Figure 8D**). Furthermore, the one-, three-, and five-year prediction calibration curves nearly coincided with the standard curve, indicating that the nomogram performed well in terms of prediction. Furthermore, decision curve analysis (DCA) was used to verify the model’s robustness, and both CSRS and nomogram yielded much more advantages than the extreme curves. Furthermore, when compared to age, both the nomogram and the CSRS had a better ability to predict prognosis (**Figures 8E, F**).

## Discussion

In clinical practice, the long-term efficacy of LGG therapy has been a significant issue due to the instability of LGG and the complexity of TME. Therefore, we need to develop and optimize the appropriate therapeutic interventions urgently. With the development of microarray technology and RNA-seq, many research studies have used gene expression profiles to categorize tumors. Predictive models according to gene expression profiles using mathematical and statistical modeling techniques have tremendous clinical potential. Cells undergo different types of senescence depending on the type of stress and/or stimulus, including stress-induced premature senescence (SIPS), oncogene-induced senescence (OIS), replicative senescence (RS), paracrine senescence (PS), treatment-induced senescence (TIS) and epigenetics-induced senescence (EIS) (24). Senescent cells collect in various organs and tissues with different physiological and pathological functions (25). Many preclinical studies prove that chemotherapy and radiotherapy cause senescent cells to accumulate in normal tissues as well as tumors. Though, senescent cells in tumors can partially stimulate metastasis, tumor recurrence, and resistance to therapy by expressing a secretory phenotype linked with aging. Moreover, senescent cells in normal tissues can worsen the side effects caused as a result of certain chemotherapies or radiation. Therefore, cellular senescence can be an important target for the treatment of cancer due to its several roles (26).

506 and 408 LGG samples were acquired from TCGA and CGGA, respectively, for this study. Based on the expression of 115 prognosis-related CSRGs, HCC samples from each cohort were divided into three subtypes, with significant differences in OS between the three subtypes. The clinicopathological, genetic, route, and immunological aspects of the three subgroups were then compared. The C1 subtype had a worse prognosis, had the largest prevalence of TP53 gene alterations, and had a significant degree of immune cell infiltration, with a large proportion of them in the G3 stage. Immunomodulatory and cell cycle pathways were also active in these patients. As a result, CSRGs may be important in the immunosuppressive microenvironment and TME. Finally, differential analysis of and LASSO found a total of six prognosis-related CSRGs, including TMSB4X, CDK6, FOXM1, IGFBP5, ITGB4, and IGFBP3.

CDK6 is a major component of the cell cycle that drives the transition from the G1 to the S phase by phosphorylating and inactivating the retinoblastoma protein (27). Activation of the YAP–CDK6 pathway may slow down the aging of the brain as well as the resulting neurodegenerative diseases (28). Dysregulated CDK6 promotes the senescence bypass during tumorigenesis and progression and its inhibition restores the senescence response in tumor cells (29). Akt/Fox M1 signaling pathway-mediated MYBL2 upregulation promotes the progression of human glioma (30) and is a probable candidate gene for molecular targeted therapy and a biomarker for glioma-related radiation therapy. In breast cancer, FOXM1 has a role in response to DNA damage, genotoxic drug resistance, and DNA damage-induced senescence (31). IGFBP-5 is elevated during cellular senescence in response to the tumor suppressor p53 activation; this mechanism mediates interleukin-6/gp130-induced PS of human fibroblasts, irradiation-induced PS of human endothelial cells, and RS of human endothelial cells independent of IGF-I and IGF-II (32). ITGB4 is a structural adhesion molecule and clears airway epithelial cells by activating the p53 pathway *in vitro* and *in vivo*, and its deficiency results in senescence (33). Interfering with the NTN4-ITGB4 connection or using inhibitors of the AKT pathway concurrently with temozolomide may protect against temozolomide-induced senescence in glioblastoma and improve therapeutic efficiency (34). IGFBP3 is a hypoxia-inducible gene that regulates multiple cellular processes, such as senescence, apoptosis, cell proliferation, and EMT (35). Domenico et al. identified IGFBP-3 as one of the genes linked with senescence genes in human gliomas (36). Though the link between the progression of TMSB4X and LGG was not reported, and it is required to explore in detail.

Based on prognosis-related SCRGs, a clinical prognostic CSRS model was developed in this work. The model exhibited great robustness and sustained prediction accuracy in independent datasets, regardless of clinicopathological features. Furthermore, this model exhibited a high prediction accuracy and excellent survival prediction power, demonstrating significant efficacy in predicting the OS of LGG patients and describing the clinical characteristics of distinct individuals. The CSRS algorithm assigned each sample a unique risk score and divided patients into different risk groups. Patients in the high-CSRS group had a worse prognosis than those in the low-CSRS group, confirming our hypothesis. Furthermore, in the TCGA–LGG and CGGA cohorts, significant differences in the distribution of CSRS were detected amongst clinicopathological feature groupings. Patients in the high-CSRS group had a considerably higher “ImmuneScore” than those in the low-CSRS group, and the expression of most immune cells differed significantly between the two groups. Cisplatin, A-443654, and Bleomycin sensitivity were also higher in the high-CSRS group.

## Conclusion

The identification of three cell senescence-related molecular subtypes helped to understand the crosstalk between cell senescence and LGG development. Cell senescence had an association with activated tumor-related pathways and immune infiltration. Cell senescence was highly associated with unfavorable prognosis, which may contribute to LGG development. High cell senescence score was significantly correlated with poor prognosis and high CSRS score. In addition, the CSRS model, a classifier, was constructed and verified. This model exhibited great robustness and stable prediction performance in independent datasets, regardless of clinicopathological features. Furthermore, this model exhibited a high prediction accuracy and significant survival prediction power, which aids in prognosis prediction and the selection of optimal treatment for patients. Overall, the synergistic effect of pro-and anti-aging therapies in cancer can be used to design novel therapeutic techniques.

## Data availability statement

The original contributions presented in the study are included in the article/**Supplementary Material**. Further inquiries can be directed to the corresponding author.

## Author contributions

All authors contributed to this present work: JC designed the study, LW acquired the data. HY and XZ drafted the manuscript, SX and QQ revised the manuscript. All authors contributed to the article and approved the submitted version.

## Funding

The present study was supported by the Natural Sciences Fund of Zhejiang Province (LGF22H160019).

## Conflict of interest

The authors declare that the research was conducted in the absence of any commercial or financial relationships that could be construed as a potential conflict of interest.

## Publisher’s note

All claims expressed in this article are solely those of the authors and do not necessarily represent those of their affiliated organizations, or those of the publisher, the editors and the reviewers. Any product that may be evaluated in this article, or claim that may be made by its manufacturer, is not guaranteed or endorsed by the publisher.

## Abbreviations

AUC, area under ROC curve; CAF, cancer-associated fibroblast; CDF, cumulative distribution function; CGGA, Chinese Glioma Genome Atlas; CSRS, cell senescence risk score; CTLs, cytotoxic T lymphocytes; DCA, decision curve analysis; DEGs, differentially expressed genes; EMT, epithelial–mesenchymal transition; ESTIMATE, Estimation of STromal and Immune cells in MAlignant Tumours using Expression data; FC, fold change; FDR, false discovery rate; GBM, glioblastoma; GSEA, gene set enrichment analysis; IC50, the biochemical half maximal inhibitory concentration; ICIs, immune checkpoint inhibitors; LASSO, least absolute shrinkage and selection operator; LGG, low grade glioma; m6A, N6-adenosine methylation; MDSCs, myeloid-derived suppressor cells; OIS, oncogene-induced senescence; OS, overall survival; PTEN, phosphate and tension homology deleted on chromsome ten; ROC, receiver operation curve; RS, replicative senescence; SASP, senescence-associated secretory phenotype; SIPS, stress-induced premature senescence; ssGSEA, single sample gene set enrichment analysis; TAMs, tumor-associated macrophages; TCGA, The Cancer Genome Atlas; TIDE, Tumor Immune Dysfunction and Exclusion; TIS, treatment-induced senescence; TME, tumor microenvironment; WHO, The World Health Organization.
