# Supplementary figures and images for: Establishment of three heterogeneous subtypes and a risk model of low-grade gliomas based on cell senescence-related genes
Source: Front Immunol. 2022 Aug 16;13:982033. doi: 10.3389/fimmu.2022.982033 (PMC9424930; doi:10.3389/fimmu.2022.982033)

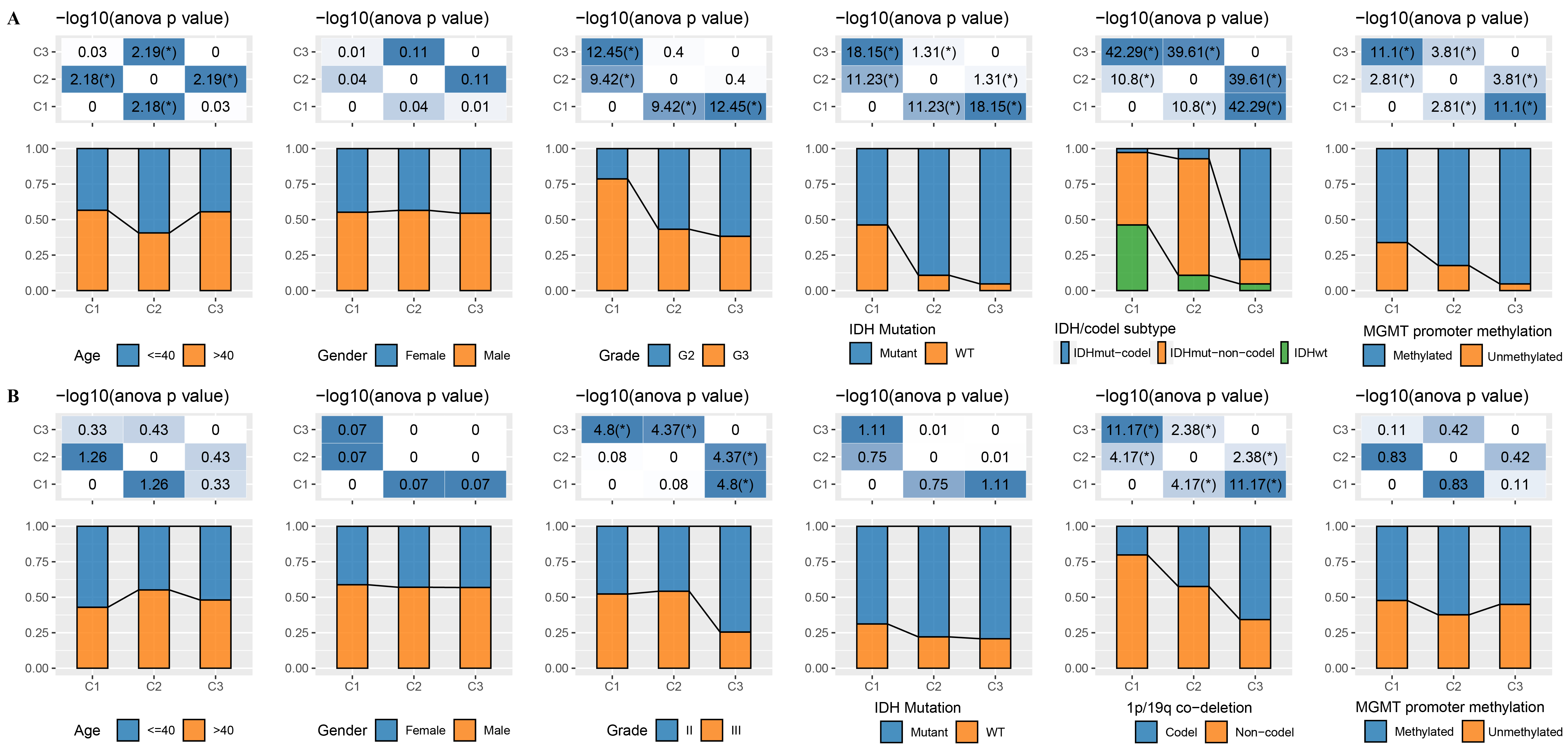

Supplement: Supplementary file 1 [file Image_1.jpeg]

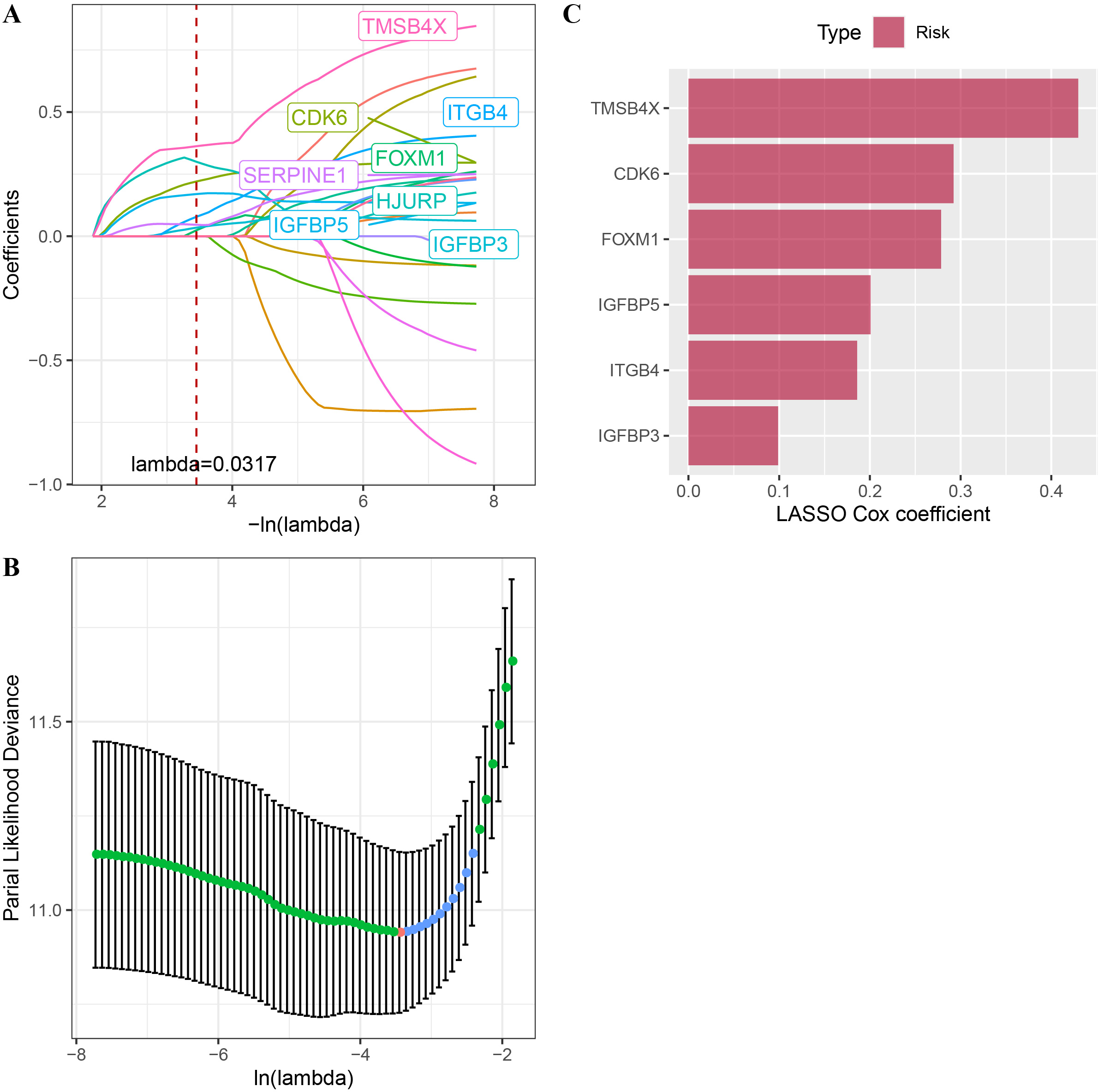

Supplement: Supplementary file 2 [file Image_2.jpeg]

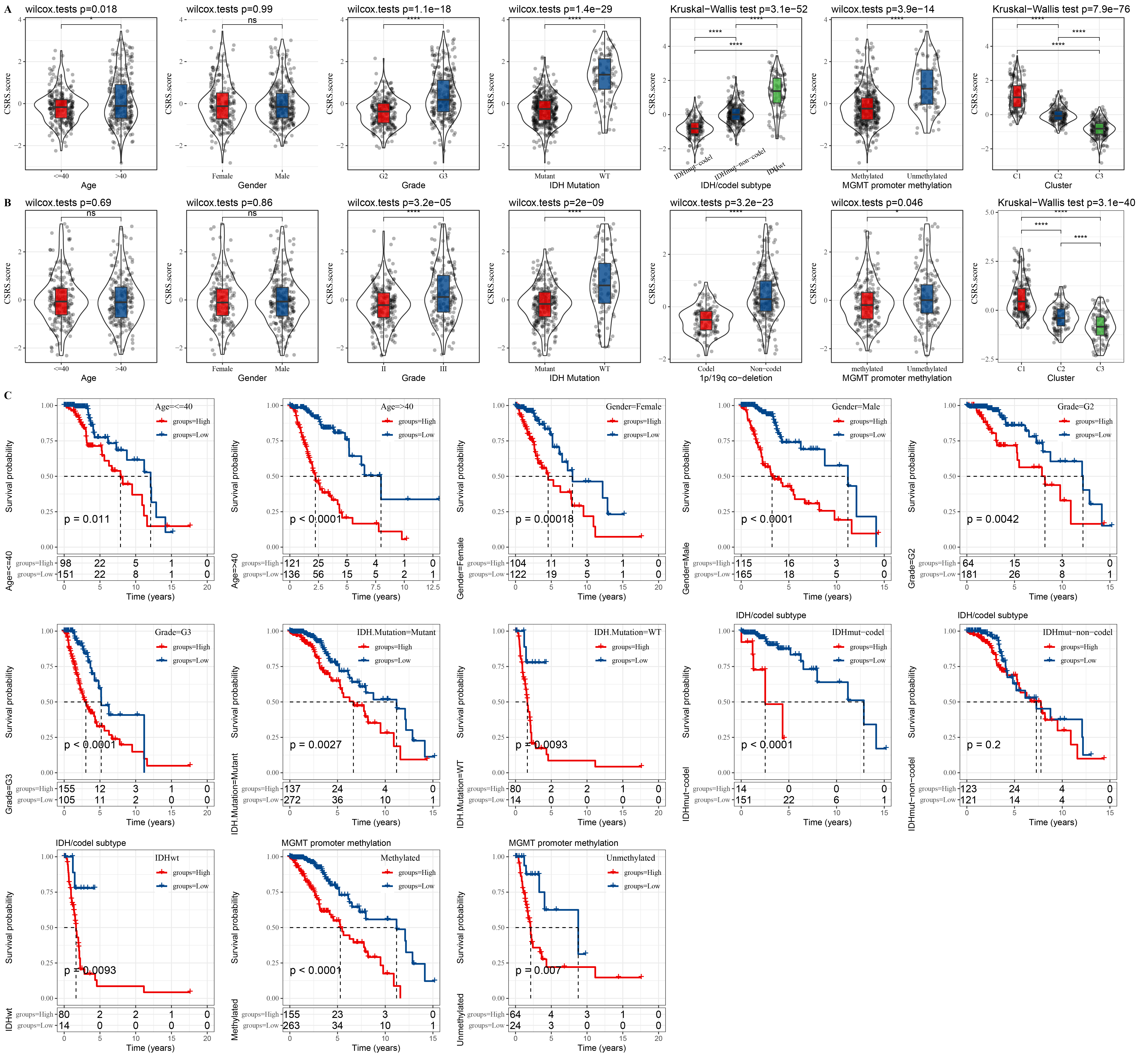

Supplement: Supplementary file 3 [file Image_3.jpeg]
